# Grapevine Root Distribution and Density in Deep Soil Layers Under Different Soil Management Practices

**DOI:** 10.3390/plants14121823

**Published:** 2025-06-13

**Authors:** Vania Lanari, Luca Pallotti, Tania Lattanzi, Oriana Silvestroni

**Affiliations:** Agricultural, Food and Environmental Sciences Department, Università Politecnica delle Marche, 60131 Ancona, Italyo.silvestroni@staff.univpm.it (O.S.)

**Keywords:** vineyard, rootstock, soil management, cover crops, soil tillage, grapevine, root length, root biomass, root system

## Abstract

Grapevine root distribution and density influence mineral and water absorption and are affected by soil management and the use of cover crops. This study, conducted in a ten-year-old commercial Mediterranean vineyard with desiccant-managed inter-rows, compares the effects of three different soil management practices—minimum tillage (MT), spontaneous natural covering (NC), and a commercial grass mixture (GM)—on root development in Montepulciano vines grafted onto Kober 5BB rootstocks. Root length, diameter, and weight across different soil layers were analyzed by digging trenches. The results show that thin roots, primarily responsible for water and nutrient absorption, ensure greater soil volume exploration, while medium-to-large roots contribute mainly to root biomass. The presence of cover crops reduces root development in the upper soil layers due to competition with herbaceous species; however, this promotes deeper root exploration and increases the total root length per plant. In the deeper soil layers, root growth is limited by higher soil compaction. Tillage enhances the development of medium-to-large roots and increases the total root biomass per plant. In conclusion, soil management influences vine root development, and competition from cover crops stimulates the growth of absorbing roots in deeper soil layers.

## 1. Introduction

Controversies over the best soil management practices in vineyards remain a pressing issue, particularly in the context of achieving high grape quality with reduced costs and minimal environmental impact [1,2]. This is especially relevant in the face of climate change, which continues to challenge traditional viticultural systems. In recent years, the use of cover crops has become increasingly widespread in vineyards, particularly in organic farming, where they are considered a promising strategy for mitigating the effects of global warming while promoting biodiversity and sustainability [1,3,4,5]. Moreover, the integration of conservation practices, such as spontaneous vegetation or cover cropping, has been shown to result in net carbon sequestration of approximately 421 g C m^−2^ [6], highlighting their role in climate change mitigation.

However, in Mediterranean regions, the adoption of cover crops is often constrained by concerns over potential competition for water and nutrients with the vine. In this context, understanding the distribution and density of vine root systems becomes increasingly important. Unfortunately, knowledge of grapevine hypogeal structures remains limited, largely due to the greater effort required for belowground studies compared to aboveground investigations [7]. Consequently, viticultural research has traditionally focused more on canopy management strategies, especially in light of their role in coping with climate change effects [8,9,10,11,12,13], all of which can impact root activity.

Roots play a fundamental role in water and nutrient uptake [14,15], the induction of specific biochemical responses [16], erosion control [17,18], and the accumulation of carbohydrate reserves [19,20,21]. This latter function becomes especially critical during budburst and early spring growth, as roots are primarily responsible for mobilizing stored carbohydrates following winter pruning, which significantly reduces woody tissues and depletes aboveground reserves [12,20,22,23,24].

Understanding how vine roots are distributed along the soil profile and how they interact with herbaceous cover is therefore essential, especially under water-limited conditions, such as during periods of summer drought. In the warmer months, vine performance in terms of yield and quality is closely tied to water uptake by the roots [25]. Among the essential nutrients, nitrogen plays a critical role in supporting vegetative growth and ensuring adequate levels of yeast-assimilable nitrogen in the berries [26].

In intercropping systems, vines and cover crops differ significantly in their root system development and soil exploration patterns. Competition for water and nutrients depends largely on root system distribution and overlap, generally inducing vine roots to penetrate deeper soil layers [27]. This competition is particularly evident in the inter-row areas, which are colonized by both species, whereas in the under-row and deeper soil layers, vine roots dominate, effectively exploiting distinct pedological zones [28].

Tillage remains a common practice to control weeds and preserve soil water and nutrients, yet it is associated with significant drawbacks, especially in hilly regions, including increased erosion and loss of soil fertility due to organic matter oxidation [29,30]. In contrast, cover cropping can mitigate erosion and facilitate tractor access, although it may increase competition for water and nutrients, sometimes leading to reduced yields [31]. Additionally, different soil management strategies can influence root development as they alter resource availability [32]. Studies have shown that permanent grass cover encourages deeper vine root systems, with a reduced root presence in the upper soil layers [27,33], suggesting that cover crops may promote deeper root growth in vines.

Moreover, root development is further influenced by environmental factors [34,35,36,37] and rootstock choice and its interaction with the scion [34,38,39,40], with most roots developing a depth between 20 and 60 cm. Agronomic practices such as high planting densities can further shape root system development, typically reducing individual root mass while increasing root penetration angle, which is associated with deeper rooting [39,41,42]. Therefore, understanding root distribution and development is essential for gaining deeper insight into the grapevine’s adaptive responses to both environmental conditions and vineyard management practices.

Given the limited knowledge available, largely due to the destructive and labor-intensive methods required for belowground investigations, especially in contrast to the extensive research on aboveground vine management, this study aims to explore how different soil management strategies influence the distribution and density of root systems in mature grapevines cultivated under Mediterranean conditions.

## 2. Materials and Methods

### 2.1. Plant Materials, Experimental Conditions, and Experimental Design

This study was conducted during the spring of 2021 in a 10-year-old hillside vineyard (~5% slope) near Ancona in the Marche region of east-central Italy (latitude: 43°32′ N; longitude: 13°22′ E; elevation: 203 m asl). Weather data were derived from the Regional Meteorological-Hydro-Pluviometric Information System (SIRMIP) provided by the Civil Protection Service of the Marche Region (Table 1).

The vineyard was planted on soil developed from fine-textured Plio-Pleistocene marine sediments, which range from sub-alkaline to alkaline (pH 7.5–8.6) and are classified as fine clayey, mixed, mesic, and Vertic Haplustept [43]. They are subjected to different soil management practices. Before the vineyard was planted, the soil had been covered with cereals for 60–70 years. The assimilable phosphorus for the vines is practically absent, falling below the minimum threshold for assimilable phosphorus, which is considered to be 23 mg/kg. More detailed soil characteristics are reported in Table 2, based on analyses carried out by external laboratories. The results clearly show that soil density increases in the deeper layers, while the availability of nutrients, specifically carbon and phosphorus, progressively decreases with depth.

The vines were planted in March 2011 with certified virus-free cuttings of Montepulciano (clone CSV-AP-MP3) grafted onto Kober 5BB rootstock. Rows were oriented from north-northwest to south-southeast, with vines planted with a spacing of 0.90 m within the row and 2.80 m between rows, resulting in a plant density of 3968 vines/ha. Vines were trained to a cordon system, vertically shoot-positioned, and pruned to leave seven spurs with two nodes each. Cordons were 0.8 m above the ground, with two pairs of catch wires providing trellising extending 0.9 m above the cordons. Pest and disease management programs followed local standard practices determined by field scouting, experience, and weather conditions. During the trial, shoots were mechanically trimmed around mid-June when their growth exceeded the top wires.

**Figure 1:**
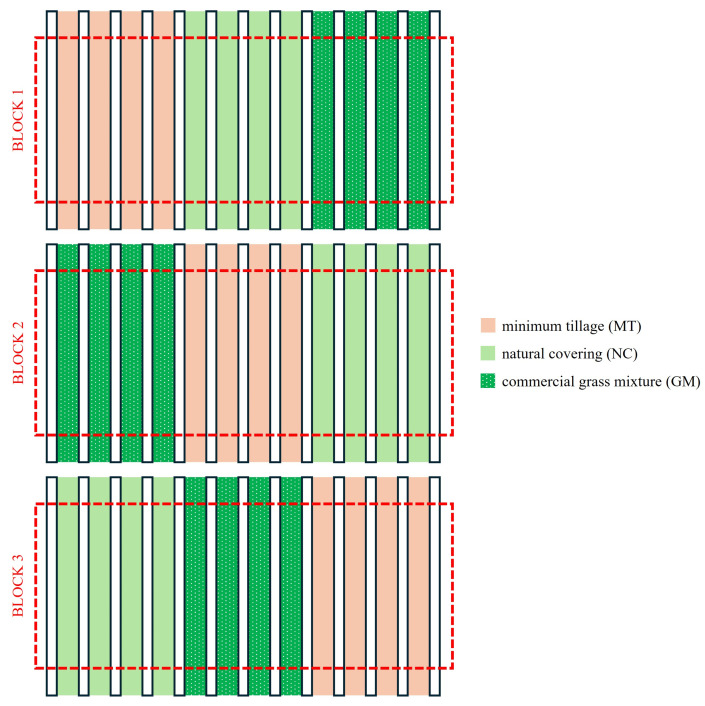
Schematic representation of the experimental design used.

**Figure 2:**
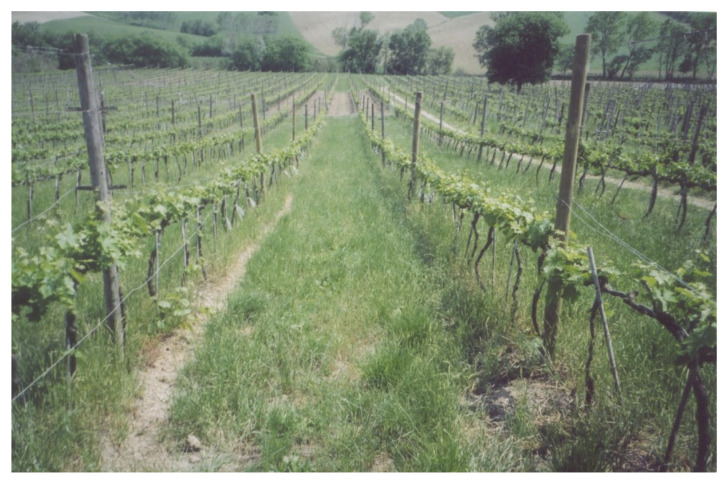
View of the inter-row area under different soil management practices.

This study was conducted in an experimental vineyard of approximately 1 ha divided into three randomized blocks, each 56 m wide and 54 m long. Each block was divided into three plots (Figure 1). The under-row was kept free of vegetation through chemical weed control over a width of 70 cm (1–2 applications per year with Glyphosate^®^), using shielding bars to prevent damage to the vines. The inter-row area has been subjected to different soil management practices (Figure 2) since 2014, including (i) periodic minimum tillage (MT), performed using a milling machine and a harrow twice per year (early spring and early summer); (ii) spontaneous natural covering (NC), with mowing repeated 3–4 times per year, allowing for the selection of naturally occurring grass species with high regrowth and tillering capacities while controlling their development, and (iii) a commercial grass mixture cover crop with low-growing grasses (45% *Festuca ovina* L., 12% *Festuca rubra* L., 24% *Poa pratensis* L., and 19% *Agrostis tenuis* Sibth.), which required three mowings per year. Starting from 2016, each year in spring, the inter-row was fertilized with 30 kg N/ha using ammonium nitrate or urea. The plots were plowed to a depth of 25–30 cm during the first three years after planting. After that, with the establishment of the different soil management practices, only superficial tillage (5–8 cm) was carried out in the tilled inter-rows, using discs, tine harrows, or small plows. In the other inter-rows, spontaneous herbaceous species were allowed to grow naturally; they were mowed twice a year, with the grass left in place.

### 2.2. Soil Excavations and Data Acquisition

**Figure 3:**
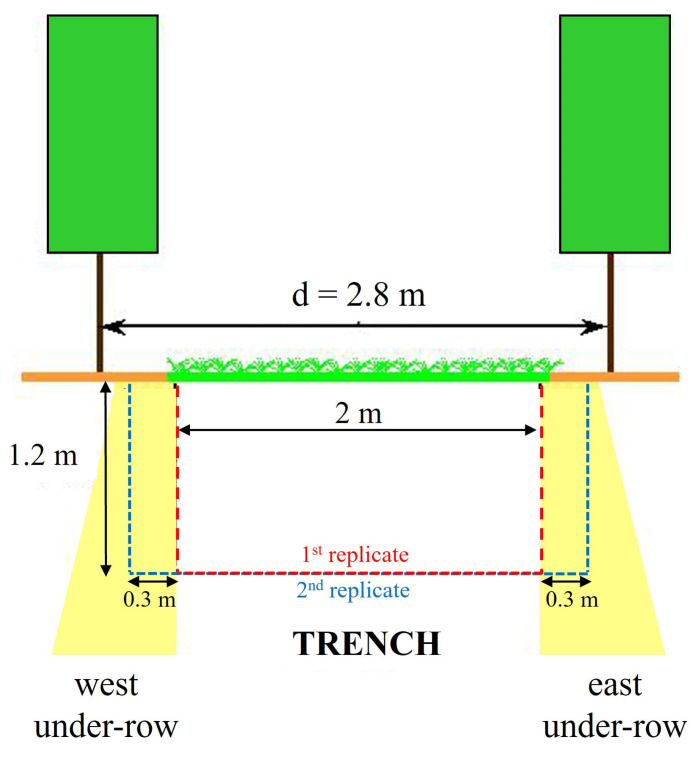
Schematic representation of the trenches.

The investigations involved inter-rows located in the center of each plot and subjected to different soil management practices, with measurements repeated in two blocks approximately 150 m apart along a gentle slope. At the beginning of spring, during the bud burst phase, when leaf photosynthetic activity and rhizodeposition were limited, a trench was opened in each inter-row, reaching a depth of at least 1–1.20 m and being approximately 2 m wide and 0.50 m long, ensuring that the area closest to the trunks was also affected (Figure 3).

**Figure 4:**
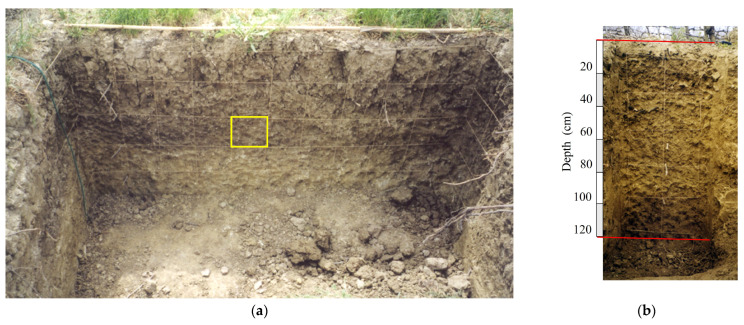
Photo of the trench profile with the 20 × 20 cm square grid system (yellow square) used for describing root distribution: (**a**) the inter-row section of the soil profile; (**b**) the under-row section of the soil profile.

Initially, a grid system with 20 × 20 cm squares was created along the soil profile on each face of the trench (Figure 4), allowing the detection and recording of root positions within each square [39]. For each square, the number and position of the roots were recorded and mapped on graph paper (scale 1:10) to determine their spatial distribution. The roots of both herbaceous plants and vines were categorized into three size classes based on their diameter (Ø):

- large (Ø > 2 mm);
- medium (1 mm < Ø ≤ 2 mm);
- thin (Ø ≤ 1 mm).

Subsequently, soil horizons were identified and coring was performed to collect four soil samples from each horizon in the inter-row profile (two from the tractor passage zone and two from the central part of the inter-row). Additionally, for each horizon, two samples were collected from the under-row section of the soil profile, west of the vines. Soil samples were taken using metal cylinders (Soil Coring Kit, Royal Eijkelkamp, The Netherlands) of a known volume (502.92 cm^3^) according to the coring method [44].

Afterward, in the laboratory, the roots were separated from the soil through washing and sieving for each soil sample. The soil samples were placed in containers with deionized water (~1.5 L per kg of sample) with a water purification system (Milli-Q^®^ A10, Millipore, Burlington, MA, USA) and left to stir overnight at an average speed of 130 rpm (Shaker KS250 basic, IKA Labortechnik, Staufen, Germany). At the end of the washing process, once the soil was fully dissolved and the roots were freed, the whole sample was poured onto a 212-micron mesh sieve for an initial separation. Then, the roots collected on the sieves were manually separated from organic residues and larger soil particles. Finally, the vine roots were separated from those of herbaceous species. The vine roots were then classified into four categories based on their diameter:

- large roots (Ø > 2 mm);
- medium roots (1 mm < Ø ≤ 2 mm);
- thin roots (0.5 mm < Ø ≤ 1 mm);
- ultra-thin roots (Ø ≤ 0.5 mm).

Each root category was then measured to obtain the length and dry weight after drying in an oven (Model NSCe180, International PBI Spa, Milan, Italy) at 110 °C until a constant weight was reached.

After soil sampling, a second replicate was performed for each trench, which was at least 0.3 m from the first, to better describe the soil profiles affected by coring and closer to the under-row (Figure 3). The spatial distribution of roots was analyzed as previously described.

The root density was determined by calculating the average values from the first and second replicates of each profile and expressed as the number of roots per m^2^. For the root density analysis, the roots were also classified into thin (Ø < 1 mm), medium (1 mm < Ø < 2 mm), and large (Ø > 2 mm) roots. Coring at different depths along the profiles and subsequent root extraction allowed for the estimation of the total root weight and length per vine and per unit of surface soil.

### 2.3. Statistical Analysis

Statistical analysis was performed using Statistica version 4.3 (StatSoft, Tulsa, OK, USA) for homogeneity of variance and subjected to ANOVA. The graphical representations were obtained using the Sigma Plot version 10 (SPSS, Chicago, IL, USA). Data were tested using means separation, calculated by applying the Student–Newman–Keuls test at *p* ≤ 0.05.

## 3. Results

### 3.1. Vine Root Distribution

The excavations, carried out between late winter and early spring 2021 (from 22 March to 15 May), before proceeding with weed containment operations, made it possible to detect, using the contact method, both the root distribution of adult vines and that of herbaceous plants, which constituted the cover crop at the time of excavation.

In minimum-tilled (MT) plots, the portion of the inter-row covered by spontaneous vegetation was around 50%. This was primarily due to the fact that the last tillage was carried out in June of the previous year, and no further intervention was performed before the trench excavation. The spontaneous vegetation primarily consisted of *Poa annua* (especially in the compacted areas of the tractor roadways), *Mercurialis annua*, *Picris echioides*, *Geranium* sp., *Veronica* sp., *Medicago lupulina*, *Sonchus asper*, and *Diplotaxis erucoides*. In the under-row, vegetation coverage was around 15% and consisted of *Veronica* sp., *Senecio vulgaris*, *Cerastium* sp., *Alopecurus* sp., *Geranium* sp., and *Picris echioides*.

In the plots with natural covering (NC), approximately 85% of the inter-row was covered by vegetation, consisting of *Trifolium* sp., *Ranunculus bulbosus*, *Geranium* sp., *Alopecurus* sp., *Bellis perennis*, *Veronica* sp., and *Picris echioides*. In the under-row, the coverage was about 40% and consisted of *Veronica* sp., *Cerastium* sp., *Alopecurus* sp., *Geranium* sp., and moss.

In commercial grass mixture plots (GM), about 80% of the inter-row was covered by vegetation consisting of *Festuca ovina*, *Festuca rubra*, *Geranium* sp., *Medicago lupulina*, *Veronica persica*, and *Alopecurus* sp. In the under-row on the west side, there was sparse vegetation (around 10%) consisting of *Ranunculus ficaria*, *Senecio vulgaris*, *Cerastium holosteroides*, *Picris echioides*, *Geranium* sp., and *Ranunculus bulbosus*, while the more sporadic vegetation covering the east under-row was represented by *Alopecurus* sp., *Geranium* sp., *Veronica* sp., *Senecio vulgaris*, and *Cerastium holosteroides*.

The trenches extended about 2 m in width and 1 to 1.2 m in depth, allowing us to trace the distribution of the roots of 10-year-old vines, which were divided into three classes based on diameter (Ø > 2 mm = large, 1 < Ø < 2 mm = medium, and Ø < 1 mm = thin). A second profile, created 30 cm from the first, allowed us to further analyze the distribution and trend of the roots. Vine roots were uniformly distributed throughout the entire trench, reaching all depths, although in the deeper layers, where the soil density is higher (Table 2), the presence of roots was sporadic and more concentrated in the center of the inter-row. In all soil profiles examined, herbaceous plants had developed their roots both in the inter-row space and in the under-row (which had been weeded for 11 months following chemical weed control treatment). The superficial soil layers were colonized by both vine roots and herbaceous plant roots, which were sometimes pushed deeper, even in the tilled plots.

**Figure 5:**
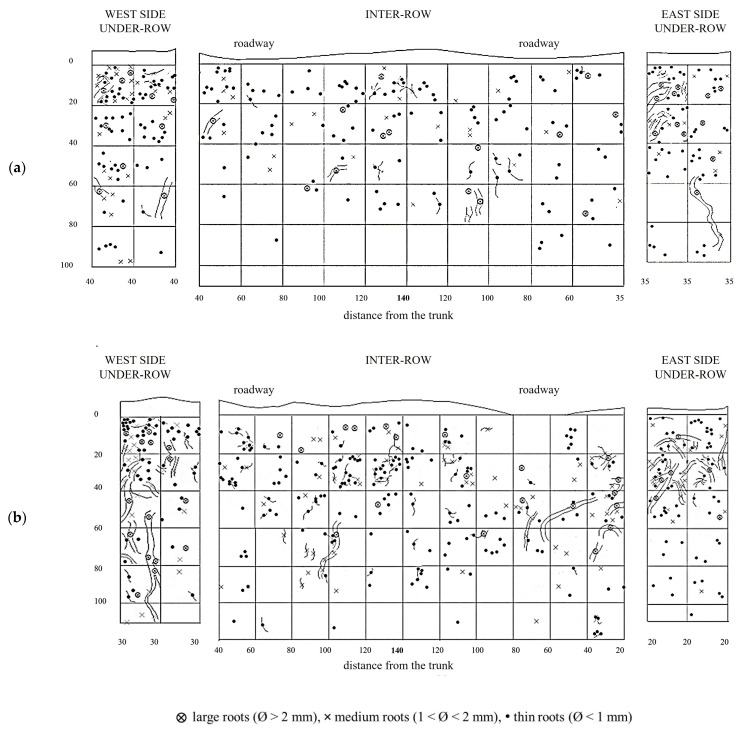
Map of soil depth and grapevine radical distribution registered in minimum-tilled (MT) plots. (**a**) First replicate; (**b**) second replicate.

MT soil profiles showed a uniform root distribution throughout the trench. Large roots (diameter >2 mm) were even found in the central area of the inter-row, which was located at the greatest distance from the trunks. Along the soil profile, the roots uniformly colonized the soil layers between 10 and 60–80 cm deep, while in deeper layers, their presence consistently decreased, almost disappearing eventually. In the weeded area of the under-row, the vine roots also colonized the first 10 cm of depth. Thin roots predominated at all depths, while medium and large roots were evenly distributed in the first 60 cm of depth. In the under-row, medium and large roots were concentrated in the first 40 cm. Note the presence of a large root placed about 60 cm deep, reaching the bottom of the profile and crossing an area characterized by the total absence of roots (Figure 5).

**Figure 6:**
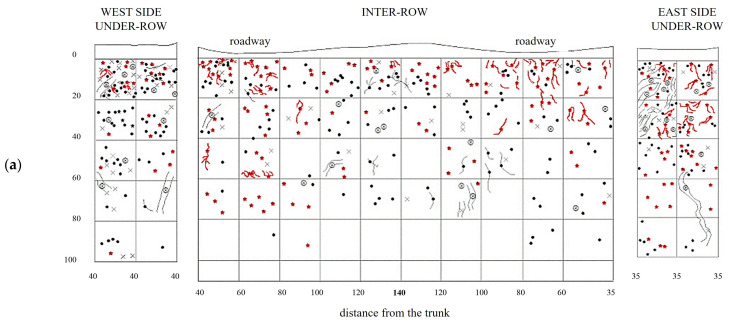

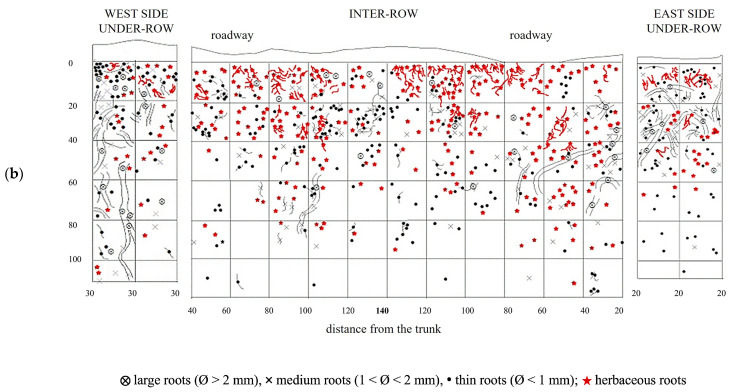
Map of soil depth and the radical distribution of grapevine and herbaceous plants registered in minimum-tilled (MT) plots. (**a**) First replicate; (**b**) second replicate.

In the under-row, the root systems of herbaceous plants developed mainly within the top 40 cm of soil, although they occasionally reached depths of up to 100 cm, particularly on the east side, where grapevine roots were less abundant (Figure 6). Similarly, in the inter-row space—periodically tilled since vineyard establishment—herbaceous roots were also primarily concentrated in the upper 40 cm. However, due to the repeated tillage that limited weed development, herbaceous roots were overall scarcely present even in the surface layers. Moreover, tillage also restricted their growth in the deeper soil horizons, where only a few roots were observed.

**Figure 7:**
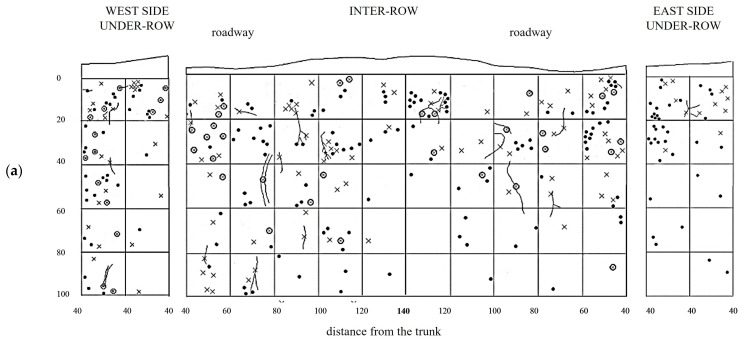

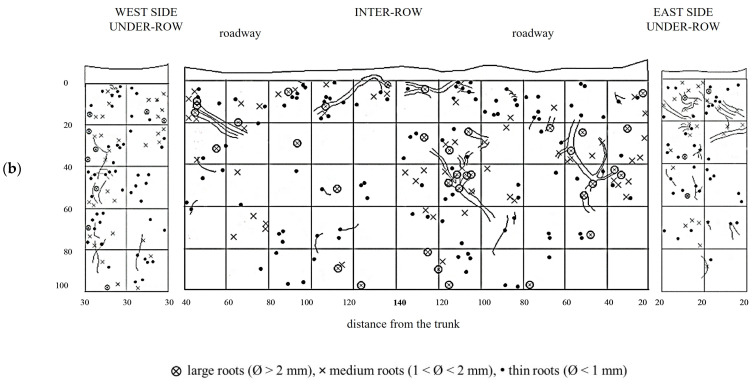
Map of soil depth and grapevine radical distribution registered in plots subjected to natural covering (NC). (**a**) First replicate; (**b**) second replicate.

NC soil profiles are shown in Figure 7. The vine roots are numerous and concentrated in the superficial layers of the inter-row, between 10 and 50 cm in depth. In the westernmost portion of the inter-row, 40–50 cm from the vine, there is a greater concentration of large roots (diameter greater than 2 mm) and a sparse presence of fine roots. On the eastern side of the inter-row, also at 40–60 cm from the vines, a high root density was observed, with a marked prevalence of fine roots. In the under-row on the west side, the root quantity is high at all depths, although the highest distribution is recorded in the first 20 cm with a high presence of large roots. In the under-row on the east side, no large roots were observed, and medium and fine roots uniformly colonized the first 40 cm of soil, followed by a reduced exploration of deeper layers.

The creation of a second profile made it possible to further evaluate the distribution of vine roots (Figure 7b). The roots are irregularly distributed throughout the entire inter-row profile. In the shallowest soil layer, corresponding to the first 20 cm, the root distribution is higher and more uniform; however, it changes as the depth increases. In particular, an area with a high density of medium and large roots can be observed in the central zone, between 20 and 60 cm in depth, while the presence of roots at the same depths tends to decrease closer to the under-row area. Along the profile, roots extend down to a depth of 100 cm, but their presence ceases in the deeper layers near the trunks. In the under-row, the root distribution is high and fairly uniform up to a depth of 0.8–1 m. Here, a significant presence of vine roots was also observed in the first 10 cm of depth.

**Figure 8:**
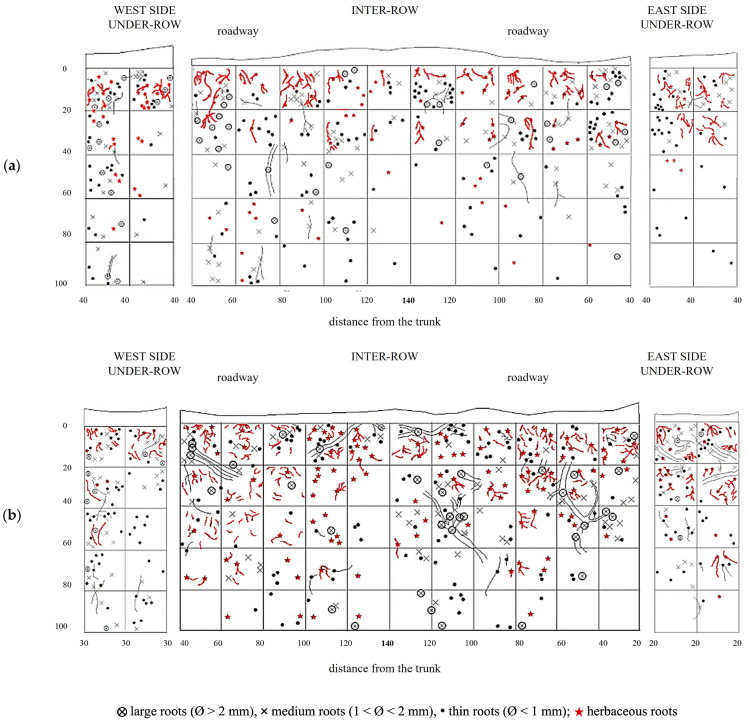
Map of soil depth and the radical distribution of grapevine and herbaceous plants in plots subjected to natural covering (NC). (**a**) First replicate; (**b**) second replicate.

Grapevine root systems in NC plots were overlaid with herbaceous plant roots (Figure 8). The first profile showed that the root systems of herbaceous plants that developed in the under-row were mainly concentrated within the top 20–40 cm of soil, although they occasionally extended beyond 60 cm. In the inter-row area, herbaceous roots were primarily concentrated in the upper 40 cm, occupying the same soil layers where grapevine roots were predominant. The excavation of the second profile confirmed that root development was mostly confined to the upper soil layers, although some deeper root growth was observed in the under-row, with traces found down to 80 cm. In the inter-row, the presence of herbaceous roots increased compared to the first profile, and although their highest concentration was still within the top 60 cm, the second excavation showed that herbaceous roots could extend to depths of around 100 cm, where vine roots remained dominant.

**Figure 9:**
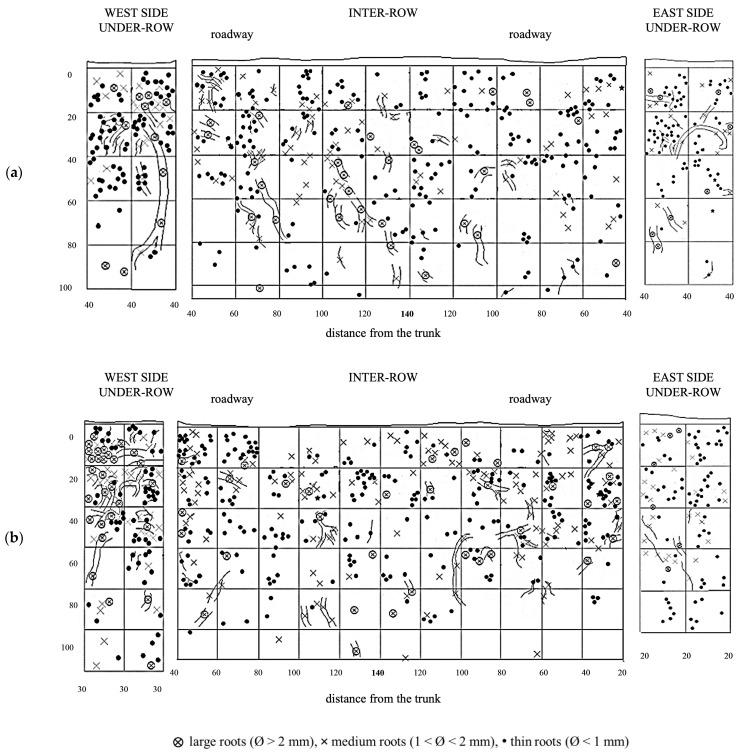
Map of soil depth and grapevine radical distribution registered in plots with a commercial grass mixture (GM). (**a**) First replicate; (**b**) second replicate.

GM soil profiles are shown in Figure 9. Thin roots are present in high quantities throughout the entire profile, although larger-diameter roots are also moderately represented. The large roots are concentrated on the west side of the inter-row and extend toward the center, reaching all depths. In the profile obtained from the under-row on the west side of the vine, thin roots are still the majority and are concentrated in the top 60 cm, below which they become very sparse. In these lower layers, a significant quantity of medium-diameter roots is present, while larger roots, though fewer, are found in the top 40 cm, one of which extends vertically into the deeper layers. In the under-row on the east side, roots are mainly concentrated in the layers from the surface down to 60 cm, although some roots, mostly large, extend down to 80 cm.

The creation of a second profile, located 30 cm from the first, allowed for further evaluation of root trends. Here, roots are uniformly distributed from just a few centimeters below the surface down to a depth of 1 m, below which only a few large-diameter roots were observed. In the west under-row, large roots are present in the first 60 cm of depth, with a notable area of high density of large roots in the top 20 cm. Thin roots are less abundant than in the east under-row. On the east side, medium and large roots are sparser but spread out to a depth of 80 cm, below which only thin roots were observed.

**Figure 10:**
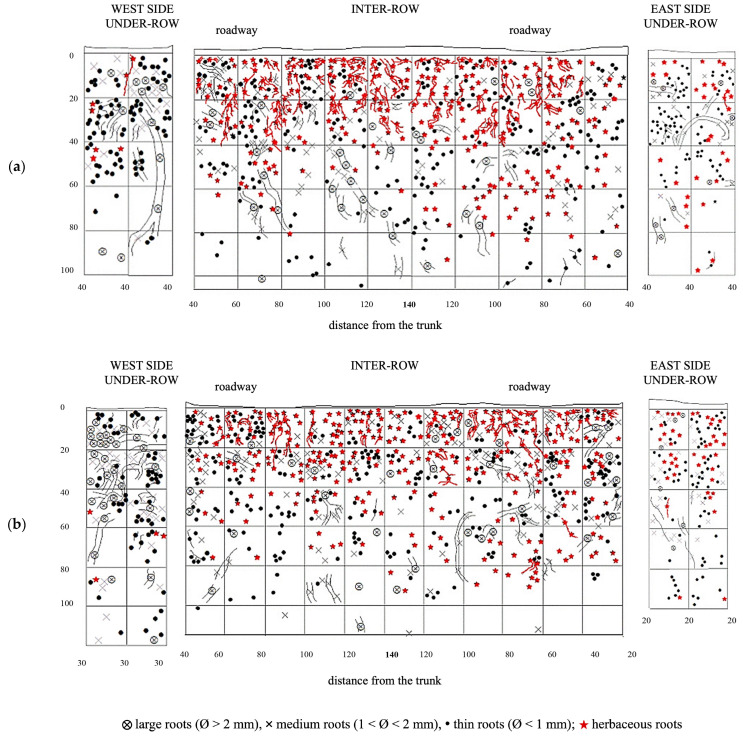
Map of soil depth and the radical distribution of grapevine and herbaceous plants registered in plots with a commercial grass mixture (GM). (**a**) First replicate; (**b**) second replicate.

The distribution of vine root systems in the GC plots was overlaid with that of the herbaceous plant roots observed in spring in the absence of management interventions (Figure 10). Among all treatments, GC plots exhibited the highest colonization by herbaceous roots, particularly in the upper soil layers. The root systems of the herbaceous plants were highly concentrated in the top 40 cm of soil but extended down to 80 cm and occasionally reached depths of up to 100 cm. Even in the inter-row area, where weeds had been chemically controlled, herbaceous roots were sporadically observed in the top 40 cm, overlapping with the root zone of the vine roots, where the latter remained dominant. The under-row on the east side showed a higher distribution of herbaceous roots compared to the west side, which is consistent with the lower presence of medium and large grapevine roots observed on this side. The second profile showed a similar distribution to the first replicate. Herbaceous roots were quite abundant in the first 60 cm of soil, but their development almost reached 100 cm of depth in the inter-row, sharing the same space with grapevine roots. In the under-row, herbaceous roots were highly present on the east-side under-row, while on the west side, they were almost absent as it was highly colonized by grapevine root systems and large-size roots.

In general, the number of vine roots was comparable to that of herbaceous plants, which accounted for 45% to 51% of the recorded root–soil contacts.

Across all examined profiles, vine roots exhibited a radial development, occupying the entire inter-row space, with a higher density in the under-row area, which accounted for 63% of the total contacts. Within this area, root density was greater on the east side of the row (33–35% of the total) compared to the west side (27–29%).

**Figure 11:**
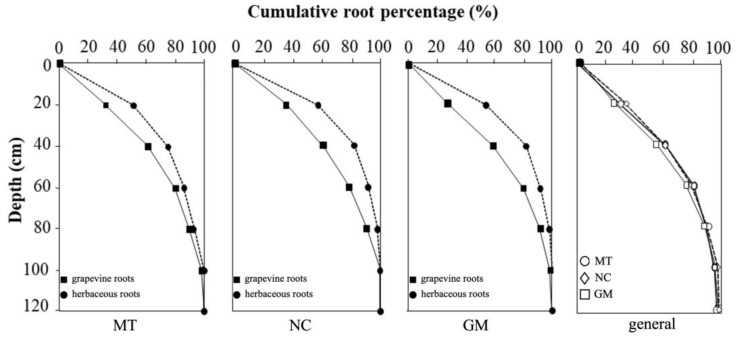
Cumulative root distribution between the inter-row and under-row of vine and herbaceous plants under different inter-row soil management techniques: minimum tillage (MT), natural covering (NC), and commercial grass mixture (GM). The final graph compares the cumulative percentage of vine root distribution under the three inter-row soil management techniques.

The data collected from each soil management treatment across the four different profiles were analyzed to describe the vertical distribution of vine and herbaceous plant root systems (Figure 11). The top 20 cm of soil showed the highest root density along the trench face, containing 39% to 45% of the total recorded roots. In this layer, on average, 53% to 56% of herbaceous roots and 27% to 36% of vine roots were observed. Within the top 40 cm of soil, 75% to 83% of the herbaceous roots were concentrated, sharing this zone with approximately 60% of the vine roots. As observed in the profile descriptions, grapevine roots became increasingly predominant at greater depths, while the percentage of herbaceous roots steadily declined.

When comparing the vertical distribution of vine roots under the three soil management treatments, greater similarity was observed between minimum tillage (MT) and natural covering (NC), both of which showed a higher concentration of vine roots in the upper soil layers. Conversely, the commercial grass mixture (GM) treatment showed a slightly lower vine root density in the topsoil but a proportionally higher contribution from deeper soil layers. This trend aligns with the herbaceous root distribution shown in Figure 10, which was notably greater in GM plots compared to the other two treatments.

### 3.2. Vine Root Density, Length, and Weight

**Figure 12:**
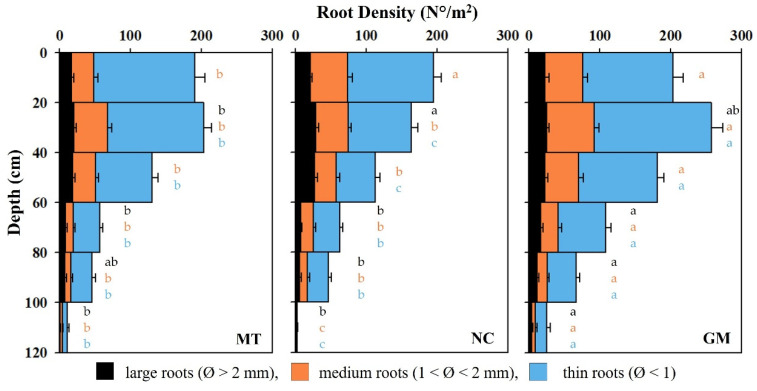
Vertical distribution of vine root density across the soil profile, categorized by root diameter (large, medium, and thin under different inter-row soil management techniques: minimum tillage (MT), natural covering (NC), and commercial grass mixture (GM)). Vertical bars represent the standard error of the mean (SE). In cases of significant ANOVA results, different letters of the same color indicate differences among root diameter classes between treatments, with a *p*-value ≤ 0.05 (*t*-test).

The vine root density, expressed as the number of roots per m^2^, is depicted in Figure 12. In the MT inter-rows, root density remained high within the first 60 cm of soil and gradually declined with increasing depth. In the top 20 cm, over 190 roots per m^2^ were recorded, with a further increase in the 20–40 cm layer, where root density exceeded 200 roots per m^2^. At 60 cm, root density decreased to approximately 135 roots per m^2^ and dropped sharply to just 11 roots per m^2^ at a depth of 120 cm. Across all layers, the root population was predominantly composed of fine roots, including in the deepest horizon (100–120 cm), where root density was the lowest. This reduction in root density in deeper soil layers can be attributed not only to depth but also to increased soil compaction, which likely restricts root penetration and development.

In the NC inter-rows, the highest vine root density was observed in the uppermost 20 cm, reaching nearly 200 roots per m^2^. Root density declined steadily with depth, falling to around 100 roots per m^2^ at 60 cm. Between 60 and 120 cm, root density decreased further from approximately sixty to just three roots per m^2^. While fine roots remained the most abundant across the profile, a substantial number of medium and large roots were observed within the top 60 cm.

In the GM inter-rows, root density was consistently higher than in the other two treatments across all depths. Root density was high at the surface and significantly increased between 20 and 40 cm, peaking at over 250 roots per m^2^. Between 60 and 80 cm, values remained relatively elevated, ranging from 180 to 110 roots per m^2^. Below 80 cm, root density progressively declined but remained above 50 roots per m^2^ at 100 cm and was still measurable at 25 roots per m^2^ at a depth of 120 cm.

**Figure 13:**
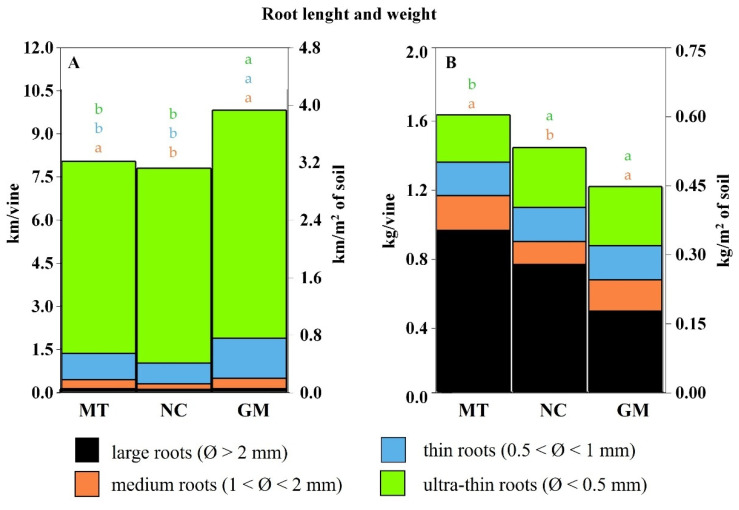
Vine root length (**A**) and dry weight (**B**), categorized by root diameter classes under different inter-row soil management techniques: minimum tillage (MT), natural covering (NC), and commercial grass mixture (GM). In cases of significant ANOVA results, different letters of the same color indicate differences among root diameter classes between treatments, with a *p*-value ≤ 0.05 (*t*-test).

Root length and dry weight are illustrated in Figure 13. Plots managed with the commercial grass mixture (GM) exhibited the greatest root length, averaging 9.8 km per vine, equivalent to 3.8 km/m^2^ of soil. Root length was slightly shorter and comparable between the minimum tillage (MT) and natural cover (NC) plots, averaging approximately 8 km per vine or 3 km/m^2^ of soil.

Regardless of the soil management technique, the contribution of medium and large roots to soil exploration was relatively limited. Instead, thin and ultra-thin roots (diameter < 1 mm) represented the majority of the total root length, accounting for 81–87% of the hypogeal system. However, these finer roots contributed minimally to the total root biomass, comprising only 17–29% of the dry weight.

In contrast to root length, root dry weight was primarily associated with medium and large roots. GM plots had the lowest root biomass, with vines averaging 1.2 kg of dry matter per plant (0.45 kg/m^2^). Vines in NC plots exhibited higher biomass at 1.4 kg per plant (0.52 kg/m^2^). The highest root biomass was observed in the MT treatment, with an average of 1.6 kg per plant or 0.6 kg/m^2^ of soil. This greater biomass allocation was largely due to a higher abundance of large-diameter roots, which accounted for approximately 58% of the total root dry weight in these plots.

## 4. Discussion

As soil depth increases, a corresponding rise in bulk density is often observed, which in turn leads to a progressive reduction in root growth. Bulk density is a critical indicator of soil compaction and physical structure, with higher values typically associated with reduced aeration, lower infiltration rates, and diminished water conductivity [45]. The limited presence of roots in deeper soil horizons can therefore be attributed to less favorable conditions for root development, including restricted water availability, poor aeration, and increased mechanical resistance due to soil compaction [46].

In the west-side under-row, roots uniformly colonized the less compact soil layers between 20 and 60 cm in depth, while in deeper layers (80–120 cm), roots were absent. On the east-side under-row, although root abundance was lower, they were still present in the uppermost soil layers.

Under Mediterranean conditions, minimum tillage combined with the use of the Kober 5BB rootstock resulted in a uniform root distribution across the entire width of the inter-row. Remarkably, roots with diameters greater than 2 mm extended even to the central part of the inter-row—the area farthest from the vine trunk—likely due to the absence of competition from weeds. However, thin roots, which play a primary role in soil exploration, remained dominant at all soil depths. Root density progressively declined with increasing depth and was nearly absent in the deepest layers, likely because of higher soil compaction, which is known to limit root development.

In the case of spontaneous natural covering, root distribution was dense, relatively uniform, and primarily concentrated within the upper 40–60 cm of soil, consistent with previous findings [47]. Nevertheless, roots were occasionally observed at depths approaching 1 m, with thin roots being the most prevalent. In the compact subsoil layers, root presence was largely restricted to soil fractures, likely due to the unfavorable, anoxic conditions commonly found at such depths [39].

In plots managed with a commercial grass mixture, the root distribution appeared to be more irregular. Roots extended across the entire surface, beginning from shallow layers (approximately 10 cm), in contrast to observations from South Africa [33] and Spain [48], where herbaceous roots were predominantly located in the top 20 cm, while grapevine roots were confined to higher depths. In the under-row, thin and medium-diameter roots generally extended to depths of up to 60 cm, while layers between 80 and 100 cm contained mostly medium and large roots. The reduced presence of thin roots in these deeper horizons may be attributed to increased soil bulk density (Table 2), which impedes their development. Nonetheless, the presence of absorptive roots indicates that grapevines can explore even greater depths. Indeed, a considerable number of roots were recorded at the lowest excavation level, at a depth of 110 cm.

These findings clearly highlight the contextual presence of both vine and herbaceous roots in the most superficial layers of grassed soils, contrasting with earlier observations [27,33,39]. The limited detection of vine roots in the surface layers reported by these authors could be attributed to methodological constraints, particularly in identifying thin or super-thin roots, which may have led to their underestimation. Additionally, environmental conditions that were less favorable for surface root development in those studies may have played a role. In the present study, soil parcels were managed by minimizing or eliminating tillage interventions to reduce erosion risks. As a result, the soil exhibited a robust level of root colonization, differing markedly from the conditions described by the above-mentioned authors, where frequent tillage likely disrupted root systems.

Regardless of the soil management strategy in the under-row areas, root distribution extended down to depths of 60–80 cm and, in some cases, as deep as 100 cm. Upon visual inspection, the soil profile revealed fractures extending to a depth of approximately 40 cm. Roots were frequently found along these fissures, suggesting that they served as preferential pathways for root growth under conditions of increased soil density.

Overall, the number of vine roots was nearly equivalent to that of herbaceous species, which accounted for 45% to 51% of the total root–soil contacts recorded. In all examined profiles, vine roots developed radially, colonizing the entire inter-row space [46]. However, the root density was highest in the under-row area, which accounted for 63% of the total contacts. This zone, spanning 0.8 m across the row and kept free of vegetation through chemical weed control, exhibited a denser concentration of vine roots closer to the trunk as a result of the reduced competition for the vines, in accordance with previous findings [39]. Notably, root presence was greater on the east side of the row (33–35% of total contacts) compared to the west side (27–29%). This asymmetry is likely due to the east side receiving more prolonged sunlight exposure because of the row’s slightly northwest-facing orientation, which leads to warmer soil temperatures that are more favorable to root development [35,36,37].

Considering the results obtained, grassing appears to slightly reduce vine root development in comparison to tillage, particularly in the superficial soil layers. This reduction is likely due to spatial competition between the root systems of grapevines and herbaceous species, even though they occupy the same zones. Nonetheless, vine roots still extended into deeper soil horizons. Notably, deeper root colonization was more pronounced in plots with inter-row grass cover (NC and GM). While our findings confirmed that grapevine and herbaceous root systems can coexist within the surface soil layers under the conditions of this trial, the presence of grasses may have promoted deeper root development in vines. This likely facilitated access to subsoil resources and may have helped reduce competition in the upper soil layers. This hypothesis is further supported by observations from the MT plots, where root density was lowest in the deeper horizons. An additional noteworthy observation is that root distribution was evident even in areas corresponding to tractor passage, suggesting that the relationship between soil compaction and root growth is not always linear or predictable.

In accordance with previous findings [39,46,49,50], we observed a significantly higher vine root density in the upper 40 cm of soil, regardless of soil management technique. In this layer, thin roots were predominant, and the average root density reached approximately 200 units/m^2^ of profile examined. Favorable conditions for root development persisted in the subsequent layer, between 40 and 60 cm in depth, where root density remained relatively high, ranging from 113 to 181 units/m^2^. Below 60 cm, however, root density progressively declined across all treatments, with roots becoming nearly absent at around 1 m deep. The highest root density (258 roots/m^2^) was recorded in GM plots, which showed the greatest development of thin roots, which are primarily responsible for the soil colonization by the root systems, specifically within the 20–40 cm soil layer. Interestingly, vines in the GM plots also exhibited the greatest root density in the deeper layers (60–100 cm), supporting the notion that, in grassed soils, grapevine roots are more likely to colonize subsoil horizons, where competition for nutrients is less intense [27,39].

For all management practices and in all profiles examined, thin roots were more prevalent than those with larger diameters, representing 57 to 68% of the total. The total root length was largely represented by super-thin roots, which, while substantially contributing to the total root length per m^2^, had a minimal influence on total weight. The total root weight is almost entirely determined by roots with medium and large diameters in particular (greater than 2 mm). For this reason, vines in grassed plots, where competition induced the greater development of thin roots to explore deeper soil layers, exhibited a higher root length but lower root biomass. In contrast, it is likely that in MT plots, the lower competition may have reduced the need to develop thin roots for exploring larger volumes of soil. As a result, vines may have invested more resources in the development of medium and large roots, leading to higher biomass compared to the other treatments.

## 5. Conclusions

Based on the results of this survey, vine root distribution and the degree of soil exploration did not show significant differences among the three management methods. Minimum tillage and natural cover exhibited nearly identical root development in the superficial soil layers. In contrast, in the commercial grass mixture plots, the dense root network formed by the cover crop appeared to encourage vines to explore deeper soil layers, where competition for nutrients is less intense. Nevertheless, vine roots also effectively colonized the upper soil layers, coexisting with the herbaceous roots. On the other hand, in tilled soils, reduced competition appeared to limit root development in deeper layers compared to covered soils.

In all three management strategies, the root systems of herbaceous plants were primarily concentrated in the first 20–40 cm of soil, sharing the same zones with vine roots.

Root density generally decreases with depth, as lower soil layers are more compacted and difficult for the roots to explore. However, compaction determined by the passage of tractors did not alter root development, as they were found even in the passageways in the inter-rows.

The vine’s capacity to explore soil volume is largely driven by thin and ultra-thin roots, which significantly contribute to total root length but require minimal biomass investment and therefore have little impact on total root weight. Root biomass is mainly derived from medium and large roots. As a result, the commercial grass mixture, which promoted deeper root exploration, led to greater root length but lower total biomass. Conversely, tillage reduced interspecies competition and soil colonization, favoring a greater investment in root biomass.

Overall, grass cover slightly limits vine root development compared to tillage. Although both vine and herbaceous roots occupy the same soil zones—primarily the upper 40 cm—vine roots were consistently observed at greater depths, where herbaceous roots were less abundant. This study shows that cover crops can be effectively integrated into vineyard soil management without significantly compromising vine root development. By reducing tillage, this practice allows for reduced erosion through the establishment of a dense surface root network. Ultimately, considering the additional benefits it provides (i.e., vigor control, reduced leaching, lower evapotranspiration, etc.), cover cropping emerges as a valuable strategy for enhancing the overall sustainability of vineyard systems.

## Figures and Tables

**Table 1:** Comparison of weather data between the 2000–2020 reference period and the 2021 study year.

	2000–2020	2021
Rain (mm/year)	750	671
Summer ^a^ rain (mm)	169	100
Average annual temperature (°C)	13	14
Average summer temperature (°C)	22	24
Amerine and Winkler index (GDD ^b^)	1800	2010

^a^ Summer was defined as the period from 1 June to 31 August; ^b^ GDD—growing degree-days (average daily temperature base of 10 °C), calculated from 1 April to 31 October for each year.

**Table 2:** Average soil characteristics at different depths for the different soil management techniques: minimum tillage (MT), natural covering (NC), and the commercial grass mixture (GM).

Depth	Apparent Density (g/cm^3^)			Organic Carbon (g/kg)			Available Phosphorus (mg/kg)		
	MT	NC	GM	MT	NC	GM	MT	NC	GM
0–20 cm	1.48	1.56	1.56	10.47	14.71	14.62	2.74	2.52	1.81
20–40 cm	1.57	1.51	1.58	7.48	7.74	7.72	0.20	0.78	0.35
40–60 cm	1.58	1.60	1.63	7.02	5.25	5.63	0.23	0.45	0.31
60–80 cm	1.63	1.60	1.64	5.64	6.12	6.57	0.29	0.49	0.36
80–100 cm	1.59	1.57	1.60	2.52	4.49	3.53	0.15	0.15	0.11

## Data Availability

Data are contained within the article.

## Abbreviations

The following abbreviations are used in this manuscript:

GM	Commercial grass mixture
MT	Minimum tillage
NC	Natural covering
SE	Standard error of the mean.
